# Supplementary material for: A very likely weakening of Pacific Walker Circulation in constrained near-future projections
Source: Nat Commun. 2021 Nov 11;12:6502. doi: 10.1038/s41467-021-26693-y (PMC8585867; doi:10.1038/s41467-021-26693-y)
Supplement: Supplementary file 1 — Supplementary Information [file 41467_2021_26693_MOESM1_ESM.pdf]

Supplementary Information

A very likely weakening of Pacific Walker Circulation in constrained near-future projections

Mingna Wu[1,2], Tianjun Zhou[1,2*], Chao Li[3], Hongmei Li[3], Xiaolong Chen[1], Bo Wu[1], Wenxia Zhang[1], Lixia Zhang[1]

1 State Key Laboratory of Numerical Modeling for Atmospheric Sciences and Geophysical Fluid Dynamics, Institute of Atmospheric Physics, Chinese Academy of Sciences, Beijing, China

2 College of Earth and Planetary Sciences, University of Chinese Academy of Sciences, Beijing, China

3 Max Planck Institute for Meteorology, Hamburg, Germany

*Corresponding author: Tianjun Zhou

Email: zhoutj@lasg.iap.ac.cn

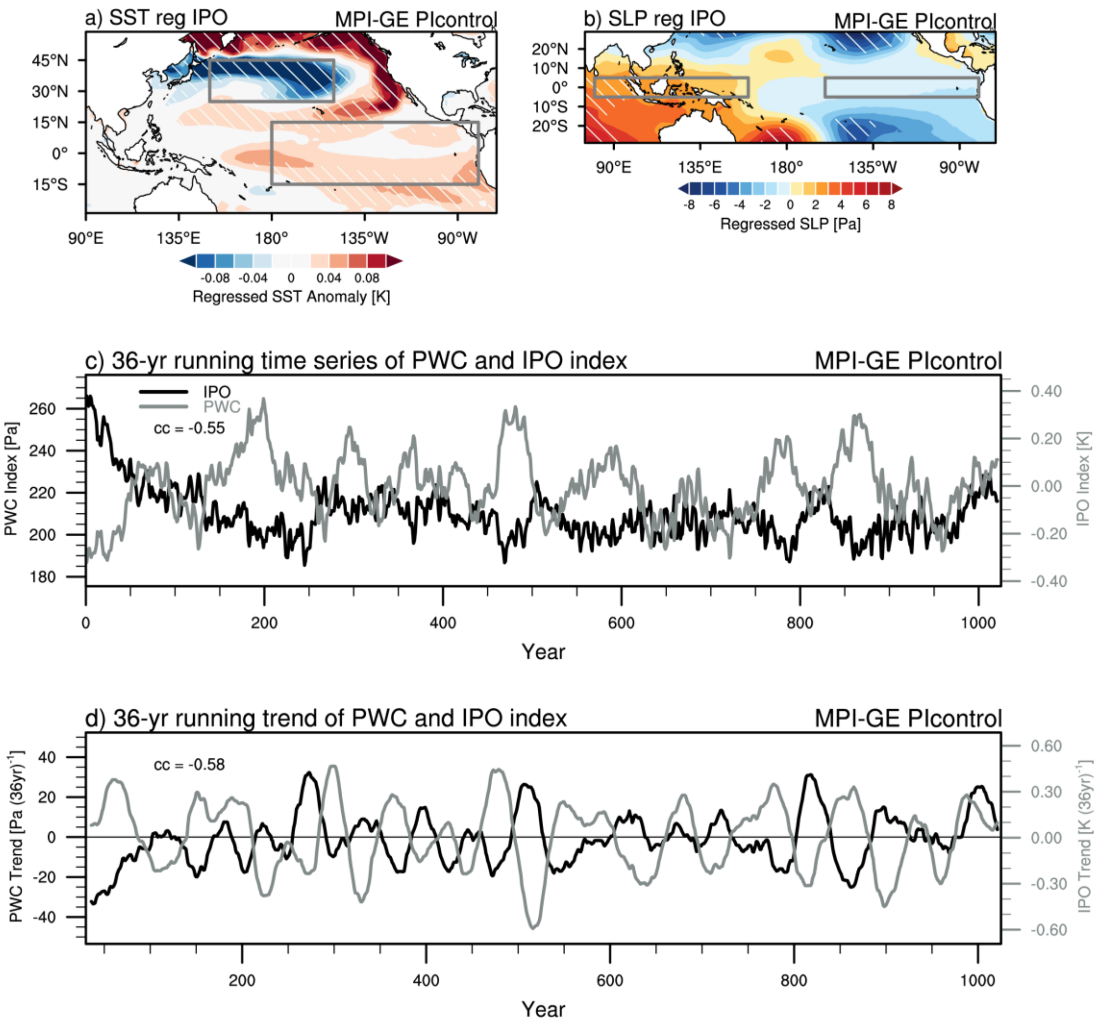

Supplementary Figure 1 | The role of IPO in modulating the PWC change in MPI-GE pre-industrial control simulation. a. The regressed SST anomalies [units: K] and **b.** SLP [units: Pa] with respect to the standardized IPO index. Rectangles in **a.** and **b.** highlight the regions used to calculate the IPO and PWC index, separately. Slant hatching denotes regressed regions significant at the 95% confidence level. **c.** The 36-year running time series of PWC index [black line, units: Pa] and IPO index [gray line, units: K]. **d.** The 36-year running trends in PWC index [black line, units: Pa (36 year)$^{-1}$] and IPO index [gray line, units: K (36 year)$^{-1}$]. The correlation coefficients (cc) are indicated at the top of the panel.

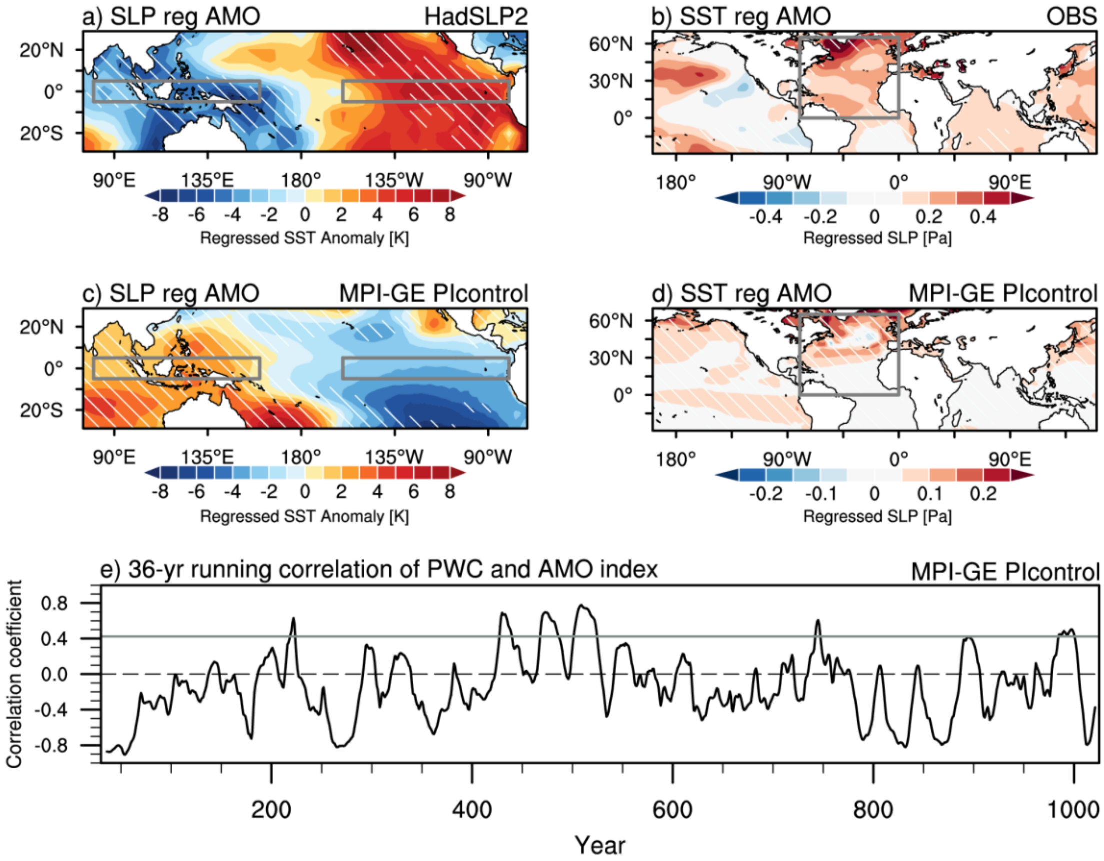

Supplementary Figure 2 | AMO-related PWC change and SST anomalies in observation and MPI-GE. a. The regressed SLP [units: Pa] from HadSLP2 and **b.** SST anomalies [units: K] from ERSST v4 with respect to the standardized AMO index during the period of 1980-2015. Rectangles in **a.** and **b.** highlight the regions used to calculate the PWC and AMO index, separately. **c-d.** The same as **a-b.** but in the MPI-GE pre-industrial control simulation. Slant hatching denotes regressed regions significant at the 95% confidence level. **e.** The 36-year running correlation between the PWC index and AMO index. The gray solid line denotes the observed correlation coefficient (cc = 0.42) between the PWC index and AMO index during the period of 1980-2015.

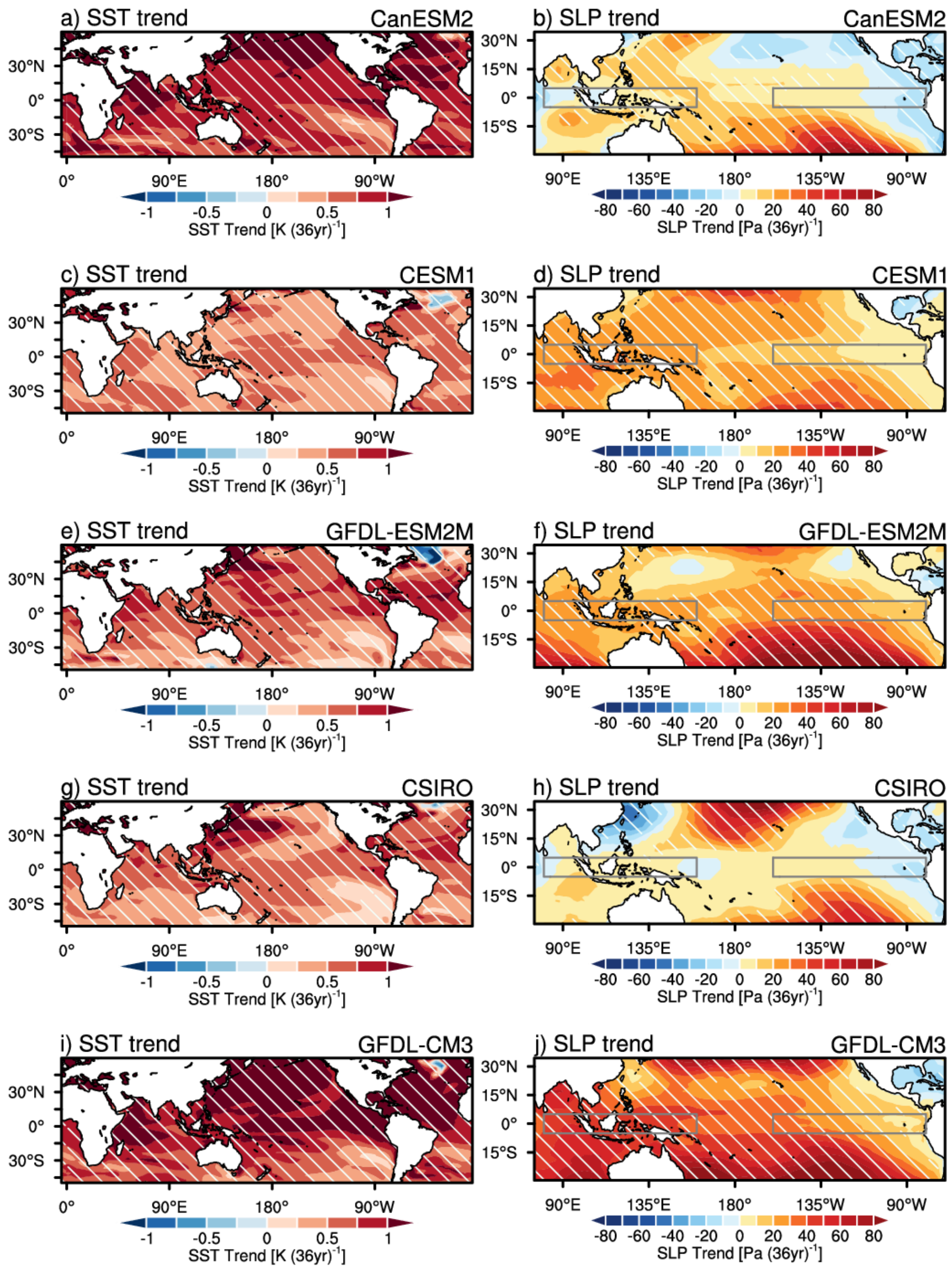

Supplementary Figure 3 | Comparison of the externally-forced SST and SLP trends during 1980-2015 among different large ensemble simulations. a., c., e., g., and i. Spatial distribution of trends in SST and **b., d., f., h., and j.** in SLP for CanESM, CESM1, GFDL-ESM2M, CSIRO and GFDL-CM3, respectively. Rectangles highlight the regions over the equatorial central/eastern Pacific (5°S-5°N, 160°-80°W) and the Indian Ocean/western Pacific (5°S-5°N, 80°-160°E). Slant hatching denotes trends significant at the 95% confidence level.

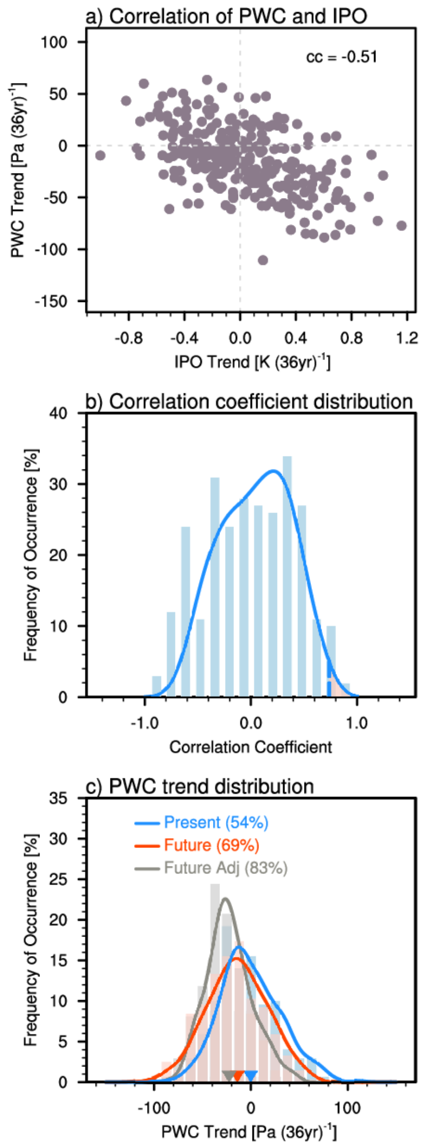

Supplementary Figure 4 | IPO constraint based on the selection of ensemble members. a. Scatter plot of projected IPO index trends [x axis, units: K (36 year)$^{-1}$] and PWC index trends [y axis, units: Pa (36 year)$^{-1}$] during 2016-2051 under the RCP8.5 scenario among all 270 realizations from six large ensembles (LEs). The correlation coefficient (cc) is indicated in the upper-right of the panel. **b.** Histograms (bars) and fitted distribution (lines) of correlation coefficients between the member-simulated and observed IPO time series during 1980-2015. The blue bars and fitted curve show the frequency of occurrence [units: %] of the correlation coefficients. Members with top 5% correlation coefficients are selected as best match members, as indicated by the red shading. **c.** Histograms (bars) and fitted distribution (lines) of PWC trends [units: Pa (36 year)$^{-1}$] derived from six LEs. The blue, red, and grey bars and fitted curves show the frequency of occurrence [units: %] of the PWC trends for present (Present), future (Future) and future with adjustment (Future Adj). The numbers in percentage indicate the percentage of members showing negative PWC trends. Differences between the adjusted and original future distributions are significant at 99% confidence level.

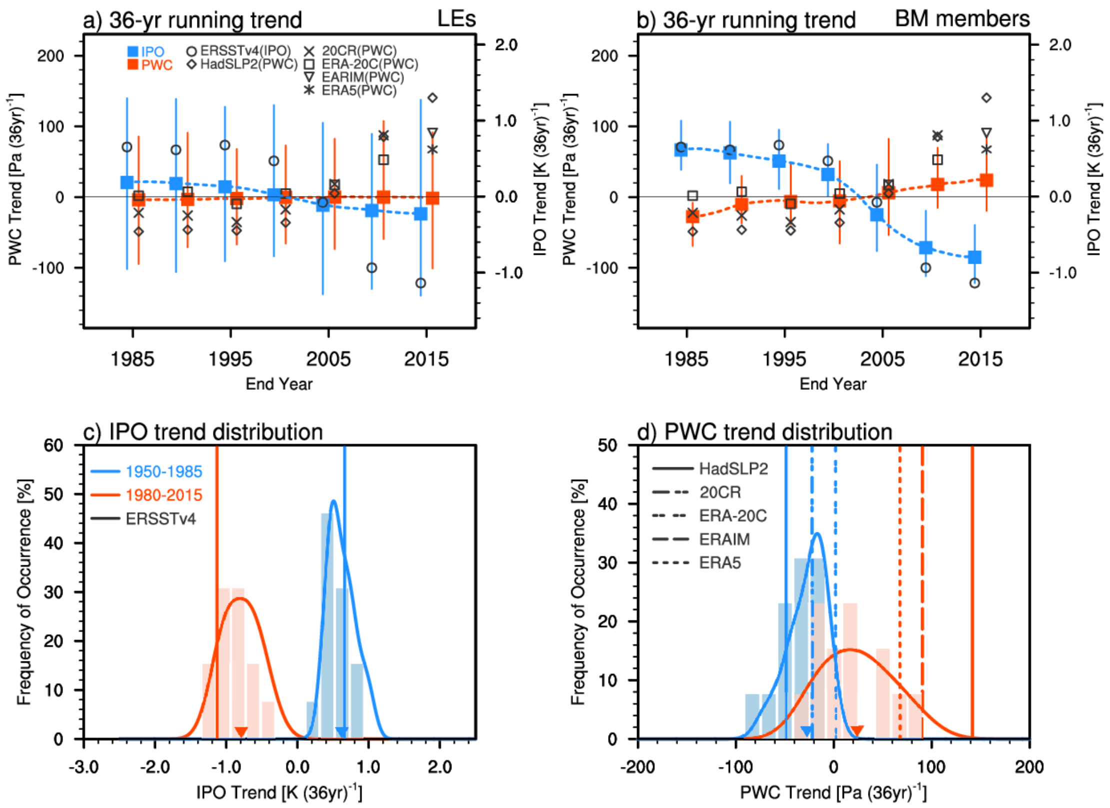

Supplementary Figure 5 | Verifying the method of selecting ensemble members according to observed IPO. a. The 36-year running trends in PWC index [units: Pa (36 year)$^{-1}$] and IPO index [units: K (36 year)$^{-1}$] during 1950-2015 obtained from six large ensembles (LEs). Dashed Lines with squares in purple (orange) and blue (red) indicate the ensemble mean IPO (PWC) index trends by LEs, with error bars representing the ensemble member spread. Black markers are form different observational and reanalysis datasets (ERSST v4 for IPO index trend; HadSLP2, 20CR, ERA-20C, ERAIM and ERA5 for PWC index trends). **b.** The same as **a.** but by the best match members (BM members). **c.** Histograms (bars) and fitted distribution (lines) of IPO index trends [units: K (36 year)$^{-1}$] derived from the BM members. The blue (red) bars and fitted curve show the frequency of occurrence [units: %] of the IPO trends for the 1950-1985 (1980-2015) period. The blue and red solid lines show the observed IPO index trend derived from ERSST v4 for the two periods. The blue and red triangles denote the ensemble mean of the distribution with the corresponding color. **d.** The same as **c.** but for PWC index trends [units: Pa (36 year)$^{-1}$] along with HadSLP2, 20CR, ERA-20C, ERAIM and ERA5. Differences between the distributions for the 1980-2015 and 1950-1985 periods are significant at 99% confidence level.

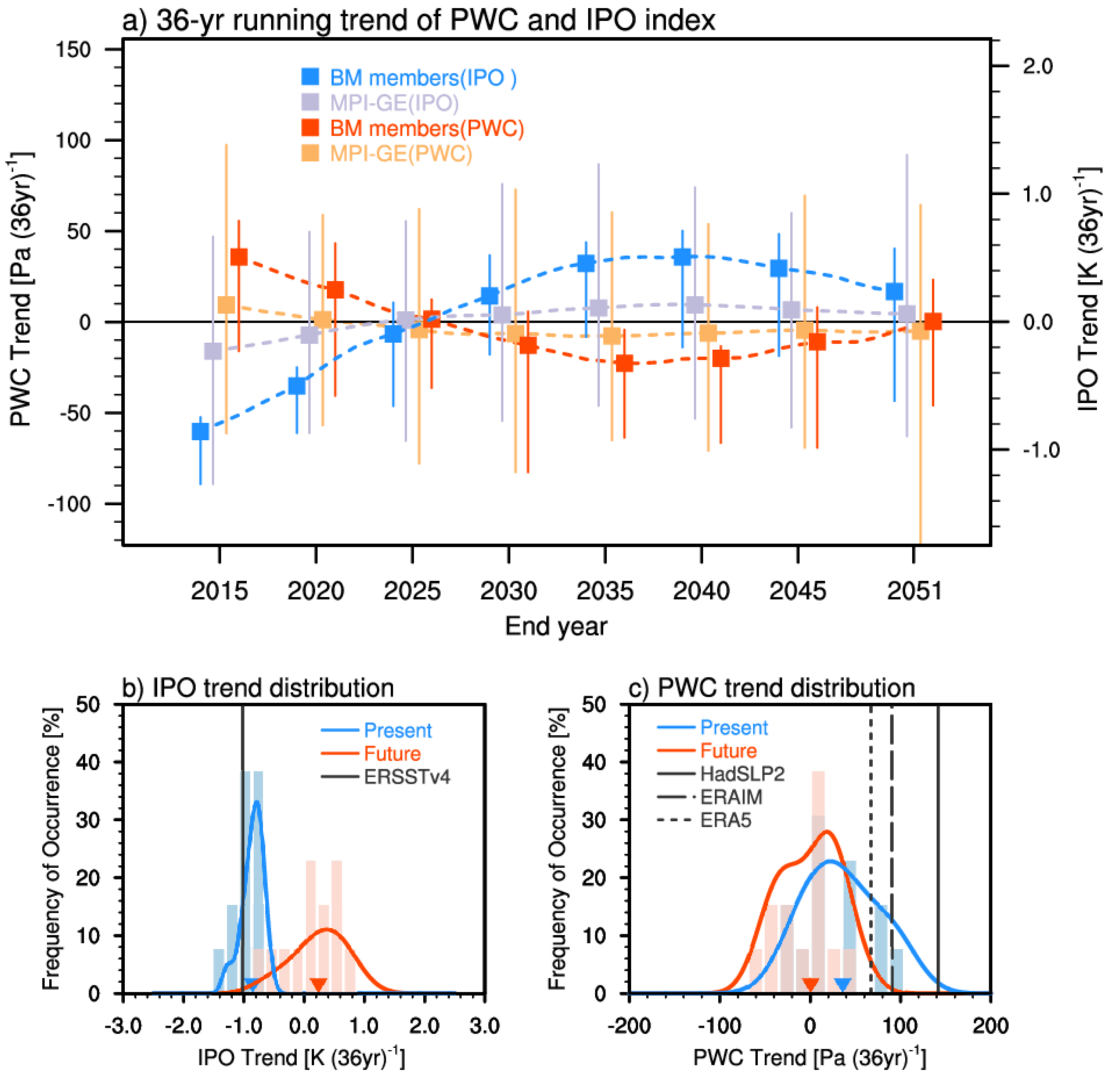

Supplementary Figure 6 | Effect of IPO on the near-term projection of the PWC under the RCP4.5 scenario. a. The 36-year running trends in PWC index [units: Pa (36 year) $^{-1}$] and IPO index [units: K (36 year) $^{-1}$] during 1980-2051 obtained from MPI-GE. Dashed Lines with squares in purple (orange) and blue (red) indicate the ensemble mean IPO (PWC) index trends by MPI-GE and the best match members (BM members) selected from MIP-GE, with error bars representing the ensemble member spread. The horizontal axis marks the end year for the 36-year segment. **b.** Histograms (bars) and fitted distribution (lines) of IPO index trends [units: K (36 year)$^{-1}$] derived from the BM members. The blue (red) bars and fitted curve show the frequency of occurrence [units: %] of the IPO index trends for present (future) climate. Black line shows the observed IPO index trend derived from ERSST v4. The red and blue triangles denote the ensemble mean of the distribution with the corresponding colors. **c.** The same as **b.** but for PWC index trends [units: Pa (36 year)$^{-1}$]. Black lines denote the observed PWC index trends derived from HadSLP2, ERAIM, and ERA5. Differences between the future and present distributions for IPO index trends and PWC index trends are significant at 99% confidence level.

Supplementary Note 1. Understanding the role of external forcing

Climate models tend to project weakened PWC under GHG forcing on the long-term timescale[1] but the role of external forcing in recent strengthening of the PWC remains controversial. This is because the balance between the forced response to aerosol forcing which tends to strengthen the PWC, and GHG forcing which tends to weaken the PWC may not be correct in climate models[2,3]. To explore the role of external forcing, we divide six LEs into two groups: Group-S (MPI-GE and GFDL-ESM2M, 130 members in total), in which external forcing induced a strengthened PWC during 1980-2015, and Group-W (CanESM2, CESM, CSIRO and GFDL-CM3, 140 members in total), in which external forcing induced a weakened PWC. We then compare the simulated and projected PWC change for both the 1950-2015 and 1980-2051 periods from those two groups (Supplementary Fig. 7). The result shows that external forcing is likely to strengthen the PWC in Group-S over the past several decades, while it tends to weaken the PWC in Group-W (Supplementary Fig. 7a and c). In the near-future, however, external forcing is likely to amplify the negative PWC trends in both LE groups (Supplementary Fig. 7b and d). The internal variability contributes roughly 64% in Group-S and 68% in Group-W to the total magnitude of the averaged 36-year running trends in the projection. Consequently, internal variability superimposes on the forced response and dominates the PWC change.

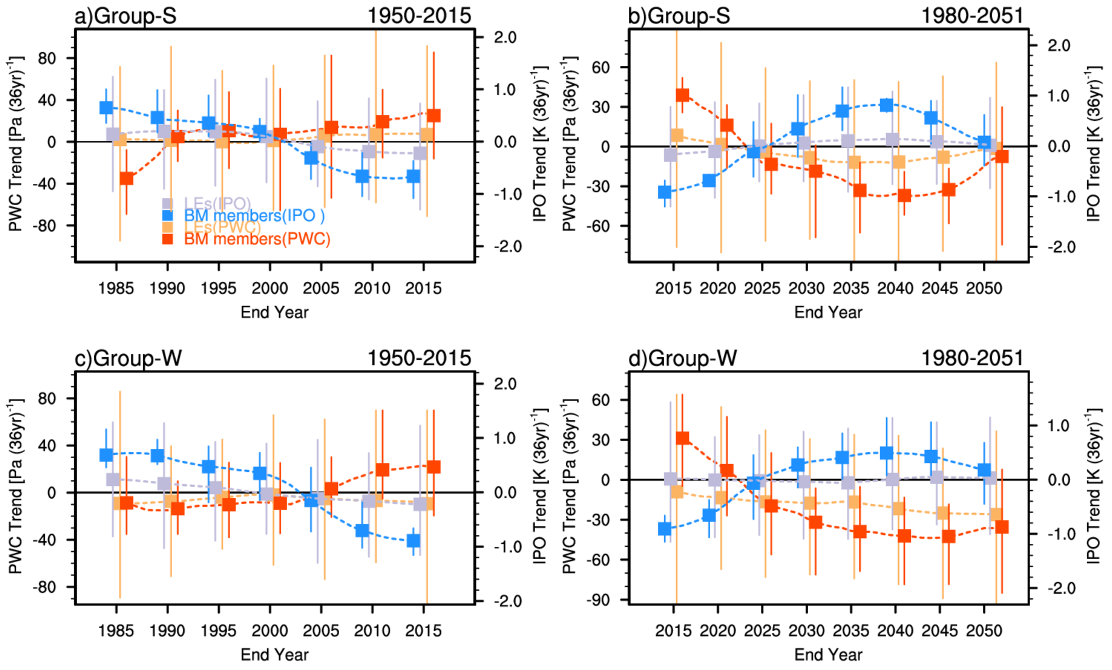

Supplementary Figure 7 | Role of external forcing. The 36-year running trends in PWC index [units: Pa (36 year)$^{-1}$] and IPO index [units: K (36 year)$^{-1}$] during (left) 1950-2015 and (right) 1980-2051 obtained from **a-b.** Group-S (MPI-GE and GFDL-ESM2M) and **c-d.** Group-W (CanESM2, CESM, CSIRO and GFDL-CM3). Dashed Lines with squares in purple (orange) and blue (red) indicate the ensemble mean IPO (PWC) index trends by large ensembles (LEs) and the best match members (BM members), with error bars representing the ensemble member spread. The horizontal axis marks the end year for the 36-year segment.

Supplementary Note 2. Model evaluation

To evaluate models' ability, we examine their performance in the simulation of the PWC change and its relation to IPO for the 1980-2015 period (Supplementary Figs. 8 and 9). Although the multimember ensemble mean of most models cannot reproduce the recent strengthening of the PWC, we find some members from the LEs can reasonably capture the observed PWC change and its relationship with IPO, with a comparable magnitude (Supplementary Fig. 8). Moreover, all the LEs are capable to simulate the negative IPO-PWC relationship (Supplementary Fig. 9).

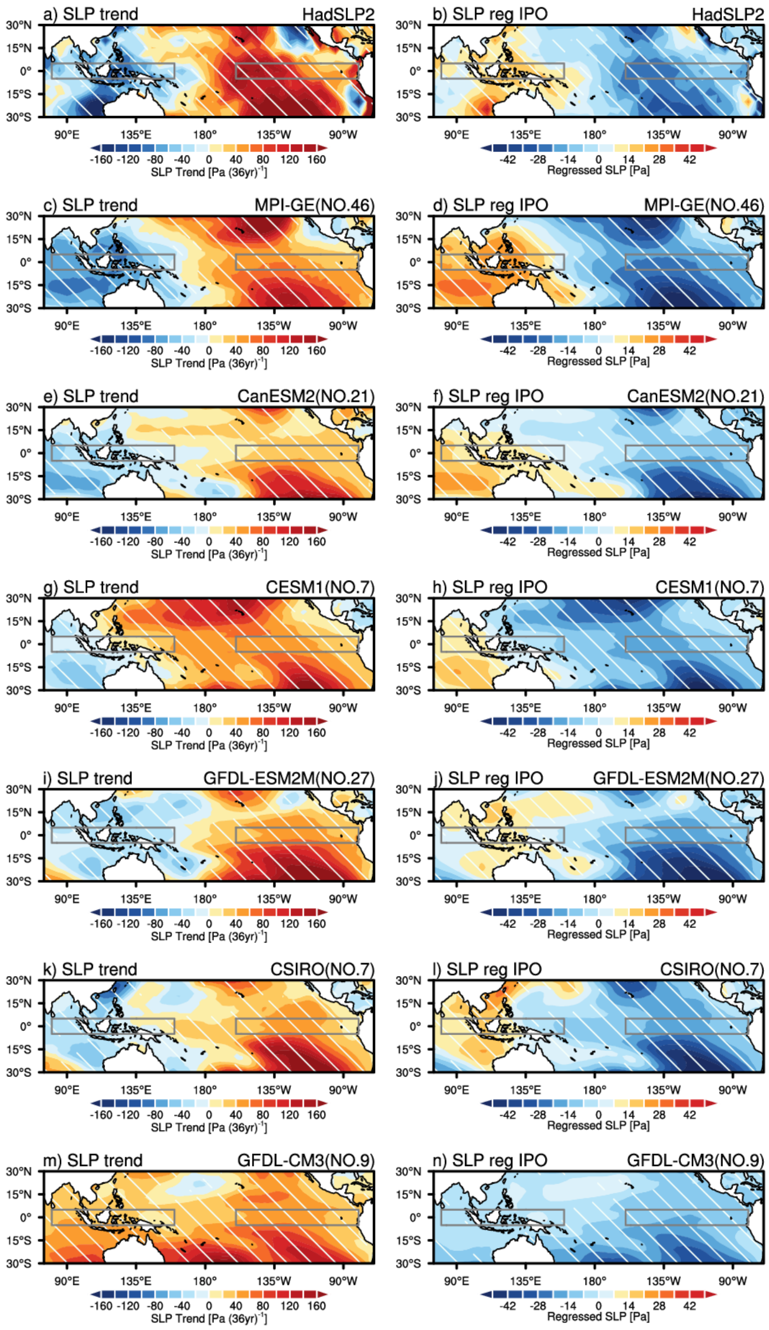

Supplementary Figure 8 | Evaluation of LEs in simulating the PWC change during the period of 1980-2015 and its relation to IPO. Spatial distribution of trends in SLP [units: Pa (36 year)$^{-1}$] derived from **a.** HadSLP2 and **c.** member 46 (NO.46) from MPI-GE, **e.** member 21 (NO.21) from CanESM2, **g.** member 7 (NO.7) from CESM1, **i.** member 27 (NO.27) from GFDL-ESMEM, **k.** member 7 (NO.7) from CSIRO, and **m.** member 9 (NO.9) from GFDL-CM3. **b.** The regressed SLP [units: Pa] from the HadSLP2 with respect to the standardized IPO index from ERSST v4. **d., f., h., j., l., n.** The same as **b.**, but for NO.46 from MPI-GE, NO.21 from CanESM2, NO.7 from CESM1, NO.27 from GFDL-ESMEM, NO.7 from CSIRO and NO.9 from GFDL-CM3. Rectangles highlight the regions over the equatorial central/eastern Pacific (5°S-5°N, 160°-80°W) and the Indian Ocean/western Pacific (5°S-5°N, 80°-160°E). Slant hatching denotes trends significant at the 95% confidence level.

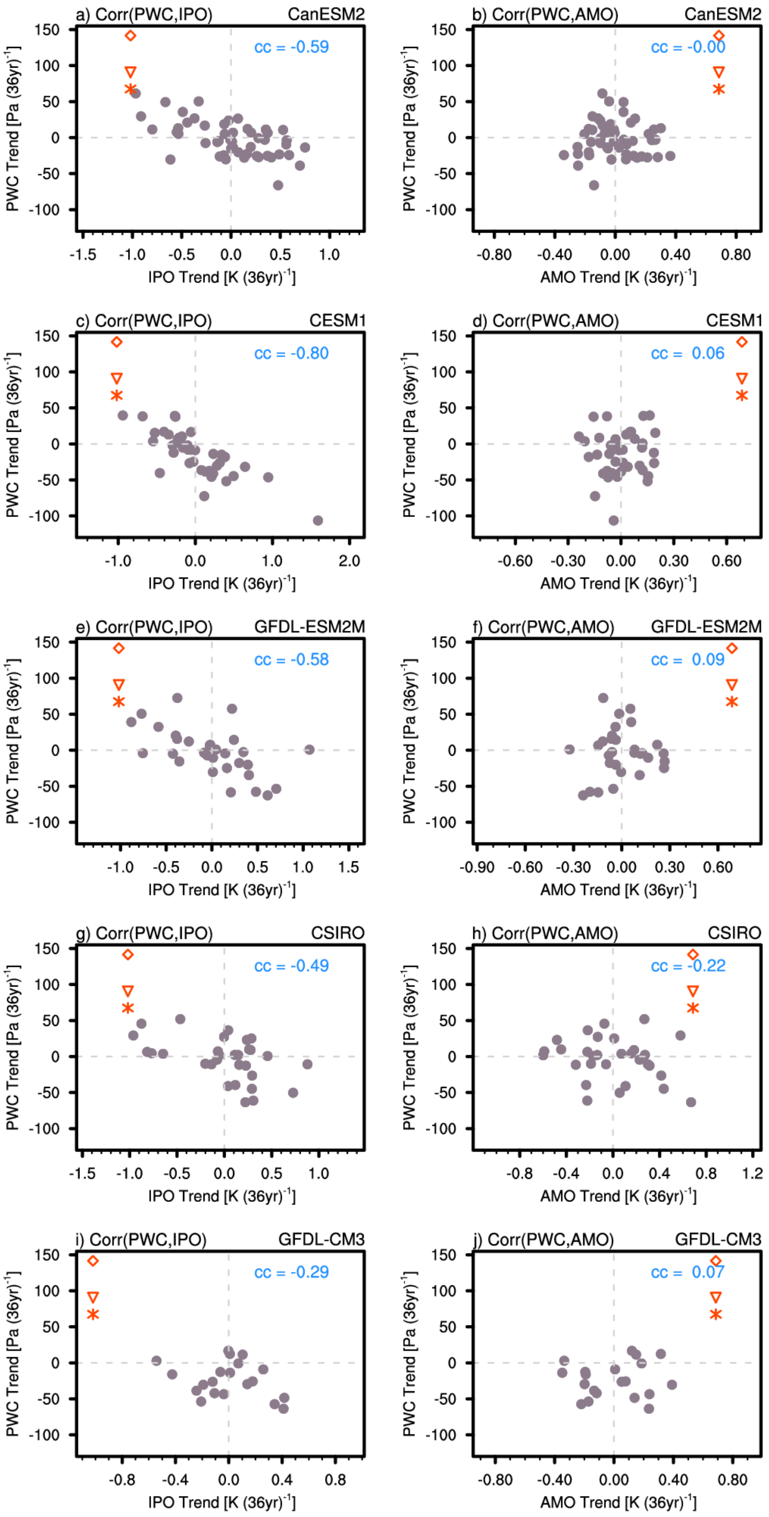

Supplementary Figure 9 | The relationship between PWC and IPO, and PWC and AMO. Scatter plot of IPO index trends [x axis, units: K (36 year)$^{-1}$] and PWC index trends [y axis, units: Pa (36 year)$^{-1}$] during 1980-2015 for **a.** CanESM2, **c.** CESM1, **e.** GFDL-ESM2M, **g.** CSIRO, **i.** GFDL-CM3, separately. Red diamond, triangle and asterisk denote the observed value from HadSLP2, ERA Interim and ERA5. The correlation coefficient (cc) is indicated at the top of the panel. **b.**, **d.**, **f.**, **h.**, **j.** The same as **a.**, **c.**, **e.**, **g.**., **i.**, but for AMO index trends.

Supplementary References

1 Vecchi, G. A. *et al.* Weakening of tropical Pacific atmospheric circulation due to anthropogenic forcing. *Nature* **441**, 73-76, doi:10.1038/nature04744 (2006).

2 DiNezio, P. N., Vecchi, G. A. & Clement, A. C. Detectability of changes in the Walker circulation in response to global warming. *Journal of Climate* **26**, 4038–4048 (2013).

3 Kociuba, G. & Power, S. B. Inability of CMIP5 Models to Simulate Recent Strengthening of the Walker Circulation: Implications for Projections. *Journal of Climate* **28**, 20-35, doi:10.1175/jcli-d-13-00752.1 (2015).
